# MYOD mediates skeletal myogenic differentiation of human amniotic fluid stem cells and regeneration of muscle injury

**DOI:** 10.1186/scrt358

**Published:** 2013-12-11

**Authors:** Ju Ang Kim, Yun Hee Shon, Jeong Ok Lim, James J Yoo, Hong-In Shin, Eui Kyun Park

**Affiliations:** 1 Department of Pathology and Regenerative Medicine, School of Dentistry, Kyungpook National University, Daegu 700-412, Republic of Korea; 2 Biomedical Research Institute, Joint Institute for Regenerative Medicine, Kyungpook National University Hospital, Daegu 700-412, Republic of Korea; 3 Wake Forest Institute for Regenerative Medicine, Wake Forest School of Medicine, Medical Center Boulevard, Winston-Salem, NC 27157, USA

## Abstract

**Introduction:**

Human amniotic fluid stem (hAFS) cells have been shown to differentiate into multiple lineages, including myoblasts. However, molecular mechanisms underlying the myogenic differentiation of hAFS cells and their regenerative potential for muscle injury remain to be elucidated.

**Methods:**

In order to induce myogenic differentiation of hAFS cells, lentiviruses for MYOD were constructed and transduced into hAFS cells. Formation of myotube-like cells was analyzed by immunocytochemistry, and expression of molecular markers for myoblasts was analyzed by reverse transcription polymerase chain reaction and Western blotting. For *in vivo* muscle regeneration, MYOD transduced hAFS cells were injected into left tibialis anterior (TA) muscles injured with cardiotoxin, and muscle regeneration was analyzed using hematoxylin and eosin, immunocytochemistry and formation of neuro-muscular junction.

**Results:**

*MYOD* expression in hAFS cells successfully induced differentiation into multinucleated myotube-like cells. Consistently, significant expression of myogenic marker genes, such as *MYOG*, *DES*, *DMD* and *MYH*, was induced by *MYOD*. Analysis of pre-myogenic factors showed that expression of *PAX3*, *MEOX1* and *EYA2* was significantly increased by *MYOD*. MYOD was phosphorylated and localized in the nucleus. These results suggest that in hAFS cells, MYOD is phosphorylated and localized in the nucleus, thus inducing expression of myogenic factors, resulting in myogenic differentiation of hAFS cells. To test regenerative potential of MYOD-transduced hAFS cells, we transplanted them into injured muscles of immunodeficient BALB/cSlc-nu mice. The results showed a substantial increase in the volume of TA muscle injected with MYOD-hAFS cells. In addition, TA muscle tissue injected with MYOD-hAFS cells has more numbers of neuro-muscular junctions compared to controls, indicating functional restoration of muscle injury by MYOD-hAFS cells.

**Conclusions:**

Collectively, our data suggest that transduction of hAFS cells with MYOD lentiviruses induces skeletal myogenic differentiation *in vitro* and morphological and functional regeneration of injured muscle *in vivo*.

## Introduction

Recently, accumulating evidence has demonstrated the ability of amniotic fluid stem (AFS) cells to differentiate into multiple lineages [1-4]. Indeed, clonal AFS cells can produce all three embryonic germ layer-derived cells, allowing for classification of AFS cells as broadly multipotent cells [5-7]. Although AFS cells originate from embryonic and extra-embryonic tissues [4], unlike embryonic stem cells, which are obtained from the inner cell mass of blastocysts, they do not form tumors after implantation in mice [7]. As a consequence, AFS cells could be a safe and easily available stem cell source for therapeutic purposes [2,6-8].

AFS cells harbor some of the characteristics of embryonic stem cells. In particular, AFS cells express the transcription factor OCT4, which is known to be expressed in embryonic stem cells. In addition, AFS cells also express the pluripotent stem cell marker, telomerase reverse transcriptase [9]. These results suggest that, in terms of their versatility, AFS cells have an intermediate potential between embryonic and adult stem cells [7,9].

Myogenic differentiation of mesenchymal stem cells (MSCs) can be induced by DNA demethylation, co-culture with myoblasts, or *in vivo* muscle engraftment [10-15]. These stimuli are associated with induction of *MYOD*, a master gene for myogenesis. MYOD is expressed at an early stage of myogenic differentiation, which, in turn, induces expression of other myogenesis-related genes, such as *myogenin*. In physiological conditions, MYOD can be phosphorylated or dephosphorylated. The phosphorylated form inhibits the DNA binding activity of MYOD homodimer, but does not affect DNA binding of MYOD-E12 heterodimers [16]. The dephosphorylated form of MYOD causes cell fusion under conditions of high mitogenesis [17]. During myogenesis of human adipocyte derived mesenchymal stem cells, phosphorylation of MYOD is increased at five days, and then shows a decrease over 14 days [18]. Translocation of MYOD from the cytosol to the nucleus is also regulated by phosphorylation [19]. Therefore, the activity and translocation of MYOD can be regulated primarily by phosphorylation. Recent studies have reported that overexpression of key transcription factors in developmental myogenesis, such as *MYOD*[20-22] and *PAX3*[23], induces myogenic differentiation in MSCs. However, in human AFS (hAFS) cells, the molecular mechanism of MYOD-induced myogenesis is not understood.

In this study, we demonstrate that expression of MYOD with a lentiviral system in hAFS cells induces formation of multinucleated myotubes. MYOD induces pre-myogenic gene expression, followed by expression of skeletal myogenic markers in hAFS cells. We also show that hAFS expressing MYOD can significantly promote *in vivo* muscle regeneration.

## Methods

### Isolation and characterization of hAFS cells

Human amniotic fluid (16 to 18 weeks of gestation) was obtained from donors at Kyungpook National University Hospital who provided informed consent. The amniotic fluid was transferred to the Joint Institute for Regenerative Medicine (JIRM): Kyungpook National University Hospital-Wake Forest Institute for Regenerative Medicine for isolation of hAFS cells. Isolation of hAFS cells and experimental procedures were approved by the Institutional Research Board of Kyungpook National University Hospital (KNUHBIO_09-1008). Briefly, amniotic fluid was centrifuged and cultured in (D)MEM high-glucose containing 10% FBS, and 1% penicillin/streptomycin (Invitrogen, Carlsbad, CA, USA) for one week, as previously described [7]. For maintenance of human AFS cells, the cells were cultured in α-MEM medium containing 15% ES-FBS, 1% glutamine, and 1% penicillin/streptomycin (Invitrogen), supplemented with 18% Chang B and 2% Chang C (Irvine Scientific, Santa Ana, CA, USA) at 37°C in a 5% CO_2_ atmosphere. Confluent hAFS cells were harvested by trypsinization for further expansion.

Expression of pluripotent markers was identified by RT-PCR using specific primers for *OCT4, SCF, GATA4, VIM, CK18* and *FGF5*. Cell surface mesenchymal stem cell markers such as CD44, CD73, CD90 and CD105 were analyzed using flow cytometry (FACS Aria, BD bioscience, San Jose, CA, USA), as described previously [24]. PE-conjugated primary antibodies were obtained from BD bioscience.

### Preparation of lentiviruses and transduction

The human MYOD or empty DNA vector was inserted into the lentiviral vector pHJ-1. To make the virus particle solution, human MYOD or empty pHJ-1 lentiviral vector was co-transfected with lentiviral packaging vectors (pHDM-Hgpm2, pRC/CMV-Rev1b, and pHDM.G) into 6 × 10^4^ cells/cm^2^ 293FT cells. The culture supernatant was collected 48 hours later and centrifuged for removal of 293FT cells and debris. They were frozen for storage in a deep-freezer. Lentivirus supernatant was thawed with water at room temperature and mixed with an equal volume of culture media. For lentiviral transduction, the viral supernatants were added to 6 × 10^3^ cells/cm^2^ hAFS cells in the presence of 8 μg/ml polybrene for 12 hours. After 12 hours, the cells were washed with PBS and changed with fresh culture media. First, hAFS cells were infected with eGFP lentivirus, and approximately more than 80% of the hAFS cells were eGFP-positive. The infected cells were then sorted using FACS Aria. The eGFP-positive hAFS cells were cultured for three days and then transduced again with MyoD or EV lentivirus. During this time period, the cells did not show any sign of cell death (data not shown).

### Reverse transcription PCR

Total RNA was extracted from cultured hAFS cells using Tri-solution (Bioscience, Gyeongsan, Korea). First-strand complementary DNA was synthesized with 1 μg total RNA using the Super Script II First-Strand Synthesis System (Invitrogen). From the 20 μl complementary DNA reaction volume, 1 to 2 μl were used for each PCR assay. The primers used in this study were as follows: *MYH*: forward, 5′-TGTGAATGCCAAATGTGCTT -3′, and reverse, 5′-GCCTTTATTTTGATCACC-3′; *MYOG*: forward, 5′-CAGCGAATGCAGCTCTCACA-3′, and reverse, 5′-AGTTGGGCATGGTTTCATCTG-3′; *MYOD*: forward, 5′-AGCACTACAGCGGCGACT-3′, and reverse, 5′-GCGACTCAGAAGGCACGTC-3′; *DES*: forward, 5′-CCTAC TCTGCCCTCAACTTC-3′, and reverse, 5′-AGTATCCCAACACCCTGCTC-3′; and *MEF2C*: forward, 5′-GGGTGGAGACCTCACGTCTG-3′, and reverse, 5′-TTATTTATCCTTTGATTCA CTGATG-3′. *PAX3*: forward, 5′-GTCAACCAGCTCGGCGGTGTTT-3′, and reverse, 5′-ATGGCACCAGGACGTATGGGT-3′, *MEOX1*: forward, 5′-AAAGGACCGAGGCGTGCAGC-3′, and reverse, 5′-CTCCTCCTGGGGCAGGCTGT-3′, *SIX1*: forward, 5′-CTTAAAGGCTACT GAGTGCGCCG-3′, and reverse, 5′-TGCGTAAAGCCAAACGACGGCA-3′, *EYA2*: forward, 5′-CGC TGCTGTGTGGACTCTGAGT-3′, and reverse, 5′-AGTGGGTGA GGTGCTGAAGGAAGGG-3′, *GLI2*: forward, 5′-TGGAATTTGGAACTGGCTTC-3′, and reverse, 5′-CCTCATTAAGGCC AAGGTC A-3′, *FOXC1*: forward, 5′-AGGAAGGCGAGAGGAGCAGAACAT -3′, and reverse, 5′-GATTGGCAGGGCAGATCACCC-3′, *FOXC2*: forward, 5′-TCCACGCCGCCTCTCTATCGC-3′, and reverse, 5′-TTGCGTCTCTGCAGCCCCTTAAT-3′. A primer set of the housekeeping gene *GAPDH* was used as an internal control. Complementary DNA was amplified using a LA Taq™ polymerase with GC buffer (Takara, Tokyo, Japan) with a total of 25 to 40 cycles. PCR products were resolved by agarose gel electrophoresis.

### Western blotting

hAFS cells were detached physically from culture dishes using a cell scrapper and sonicated in RIPA buffer (50 mM Tris–HCl pH 8.0, 150 mM NaCl, 1% NP-40, 0.5% sodium deoxycholate, 0.1% sodium dodecyl sulfate (SDS)). Protein concentration was determined using a BCA protein assay kit (Interchim, Montlucon, France). Protein samples were separated in SDS-PAGE and transferred to Protran membranes (Whatman, Florham Park, NJ, USA). The membrane was blocked with 3% non-fat dry milk in TBS-T and each primary and corresponding secondary antibody was incubated for one hour. Primary antibodies and dilutions used were as follows: mouse monoclonal anti-MyoD (BD biosciences) at 1:500; rabbit polyclonal anti-Myf5 (C-20) (Santa Cruz Biotechnology, Inc. Dallas, TX, USA) at 1:200; mouse monoclonal anti-desmin (BD biosciences) at 1:500; rabbit polyclonal anti-dystrophin (Abcam Inc., Cambridge, MA) at 1:200 and mouse monoclonal anti-FLAG M2 (Sigma-Aldrich Co. St. Louis, MO, USA). Secondary antibodies conjugated to horseradish peroxidase (HRP) were obtained from Invitrogen. The signal was detected using WesternBright ECL (Advensta, Menlo Park, CA, USA).

Nucleus and cytoplasm were fractionated as described previously [25]. Briefly, collected cells were re-suspended with buffer A (10 mM HEPES pH 7.9, 1.5 mM MgCl_2_, 10 mM KCl, 0.5 mM dithiothreitol (DTT), 0.05% NP40), placed on ice for 10 minutes and centrifuged at 4°C at 3,000 rpm for 10 minutes. Supernatant was kept as a cytoplasmic fraction. The pellets were resuspended in 374 μl of buffer B (5 mM HEPES pH 7.9, 1.5 mM MgCl_2_, 0.2 mM ethylenediaminetetraacetic acid (EDTA), 0.5 mM DTT, 26% (v/v) glycerol) and 26 μl of 4.6 M NaCl (300 mM NaCl). The re-suspended pellets were homogenized with full strokes in a Dounce or glass homogenizer and placed on ice for 30 minutes, followed by centrifugation (14,000 rpm) at 4°C for 30 minutes. The supernatant was used as nuclear fractions.

### Immunostaining and H&E staining

Cells plated on cover slips were fixed with 4% paraformaldehyde-PBS, and permeabilized with 0.25% Triton X-100 for MYOD, desmin, α-actinin staining. Nonspecific reactions were blocked with 3% normal goat serum. Cells were then incubated with mouse monoclonal anti-MyoD (BD Bioscience), mouse monoclonal anti-desmin (BD Bioscience) and mouse monoclonal anti-α-actinin (BD Bioscience) primary antibodies, at the dilutions recommended by the manufacturer, overnight at 4°C, followed by incubation with secondary antibodies for one hour at room temperature. Anti-mouse Alexa Fluor 488-conjugated secondary antibodies (Invitrogen) and 0.1 μg/ml of DAPI (Santa Cruz Biotechnology, Inc.) were used for immunofluorescence. Cover slips were mounted on slides using fluorescent mounting medium (Dako, Carpinteria, CA, USA).

Muscle tissues were fixed with 4% paraformaldehyde-PBS for 30 minutes at 4°C. The tissues were cryostat sectioned (10 μm thick) and permeabilized with PBS containing 0.25% Triton X-100 for five minutes. The sections were then blocked with 3% normal goat serum in PBS, and incubated rabbit polyclonal anti-dystrophin (Abcam, Cambridge, UK) at 1:50 to 1:100 dilutions overnight at 4°C. The tissue sections were washed with PBS and then incubated with TRITC-conjugated anti-rabbit immunoglobulin G (IgG) antibodies (Sigma-Aldrich) at a 1:500 dilution for one hour at room temperature. The sections were washed with PBS and mounted with fluorescent medium (Dako) with DAPI. Immunofluorescence was visualized using an LSM 5 fluorescence microscope (Zeiss, Jena, Germany).

For hematoxylin/eosin (H&E) staining, the muscle samples were fixed with 4% paraformaldehyde-PBS at 4°C for two days. The tissues were embedded with paraffin and sectioned with 5 μm thickness. The sections were stained with H&E staining using a standard protocol.

### Human AFS cells transplantation in mice

All animal experiments were performed under the guidance of the Institutional Ethics Committee of Kyungpook National University. Immunodeficient BALB/cSlc-nu mice were maintained under conventional housing conditions using a chamber system. Male mice aged 12 weeks were used. hAFS cells at passage 11 were used for *in vivo* injection. Before injection, hAFS cells were transduced with eGFP-lentivirus, and then eGFP-positive hAFS cells were sorted by fluorescence activated cell sorting (FACS). The eGFP-positive hAFS cells were cultured for three days and transduced again with empty vector- or MYOD-lentivirus. The cells were trypsinized with 0.25% trypsin/EDTA (Invitrogen) and washed with DPBS (Wellgene, Daegu, Korea). MYOD transduced hAFS cells were injected into the left tibialis anterior (TA) muscles of mice two days after lentivirus infection. Efficiency of lentivirus infection was confirmed with Western blot using MYOD antibody (Ab) as well as eGFP transduction rate. The transduction rate was approximately 80% of infected hAFS cells. BALB/cSlc-nu mice were anesthetized with intramuscular injection of 80 μl of a solution (33.8% Zoletil and 6.5% Rompun in PBS). At the point of transplantation, the left TA muscle was injected with 40 μl of 10 μM cardiotoxin, and approximately 500,000 MYOD- or EV-hAFS cells were injected using a 300 μl insulin syringe to the center of the muscle. The mice were euthanized at 7 and 21 days. The TA muscles were cross cryostat sectioned and stained with H&E and immunofluorescence. To analyze morphological changes in MYOD-hAFS cell-injected TA muscle, we examined three mice in each group. A total of 144 sections per each group (48 per mouse) were stained with H&E. For immunohistochemistry (IHC) with dystrophin, forty eight sections from each group around the center area were used, and the IHC images of dystrophin were used to calculate an average area of muscle fibers and percentage of centrally nucleated myofibers. The average size and percentage of centrally nucleated muscle fibers were measured with i-solution image software (Daejeon, Korea).

## Results

### Characterization of hAFS cells

**Figure 1:**
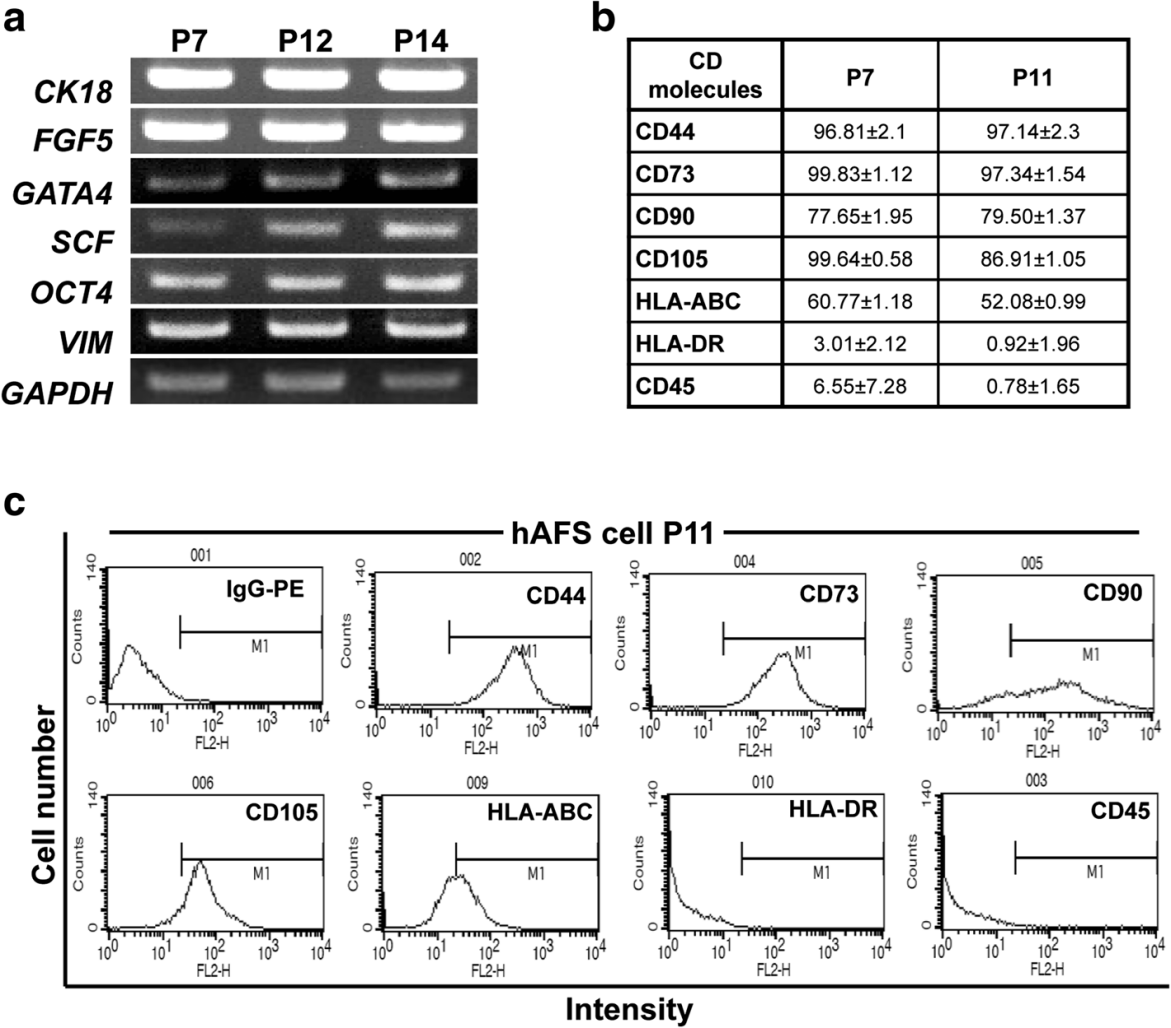
**Expression and immunotyping of stem cell markers in hAFS cells using RT-PCR and flow cytometry. (a)** RT-PCR analysis showed that expression of various stem cell markers was sustained over the population doublings. **(b)** hAFS cells expressed high levels of mesenchymal stem cell markers, including CD44, CD90, CD73 and CD105; however, they did not express HLA-DR and CD45 at passages 7 and 11. **(c)** FACS plots for MSC markers of **(b)**. hAFS, human amniotic fluid stem; MSC, mesenchymal stem cells.

Human amniocentesis specimens were obtained from Kyungpook National University Hospital. hAFS cells were isolated as described in the Methods section. In order to characterize the cells derived from amniotic fluid, we first analyzed immunophenotypes and gene expression profiles. RT-PCR analysis showed that hAFS cells express stem cell marker genes for *SCF, GATA-4, VIM, CK18, OCT4* and *FGF5* throughout the culture period (Figure 1a). These results demonstrate that cells isolated from amniotic fluid have typical expression patterns of hAFS cells [26]. In addition, we also analyzed cell surface markers of MSCs (CD44, CD73, CD90, and CD105) and major histocompatibility (MHC) antigens (HLA-ABC and HLA-DR) in isolated hAFS cells. The results showed strong expression of CD44, CD73 and CD105, and CD45 and HLA-DR were negative for these cells (Figure 1b). Expression of stem cell markers was maintained during the culture period up to passage 14 (Figure 1b and c). Collectively, these results suggest that hAFS cells express many stem cell-associated markers and those markers are well maintained during *ex vivo* expansion.

### Formation of myotube-like cells by MYOD transduction

**Figure 2:**
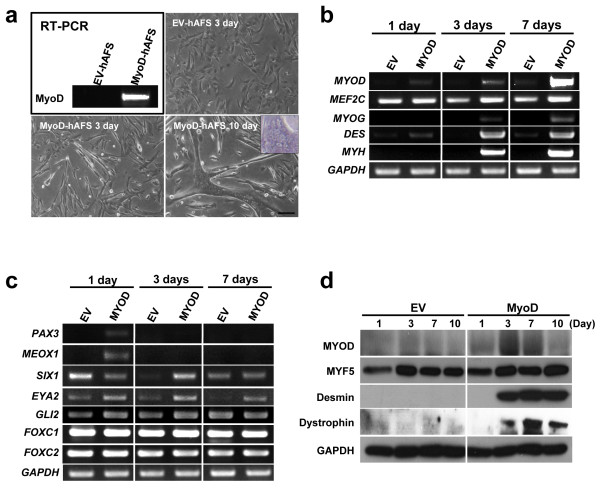
**Transduction of MYOD and EV lentiviruses to hAFS cells. (a)** MYOD mRNA expression in hAFS cells infected with empty or MYOD lentivirus was analyzed. Morphology of hAFS cells infected with EV lentivirus at day 3 and MYOD lentivirus at days 3 and 10 is shown. Myotube-like morphology was clearly detected in hAFS cells transduced with MYOD at day 10. **(b)** and **(c)** mRNA expression of myogenic genes. hAFS cells were cultured for one, three, and seven days in myogenic differentiation medium. **(b)** Myogenic marker genes were analyzed using RT-PCR. **(c)** Expression of pre-myogenic marker genes was analyzed. **(d)** Levels of myogenic proteins during myogenic differentiation of hAFS cells. hAFS cells differentiated for one, three, seven and ten days in myogenic medium. The protein levels were analyzed with antibodies specific to each myogenic protein (Scale bar = 20 μm). EV, empty vector; hAFS, human amniotic fluid stem.

To induce myogenic differentiation of hAFS cells, MYOD lentivirus was constructed and transduced into hAFS cells. An empty lentiviral vector (EV) was used as a negative control. Two days after viral transduction, hAFS cells transduced with MYOD lentivirus expressed significant amount of MYOD (MYOD-hAFS cells), while no MYOD expression was detected in empty vector transduced cells (EV-hAFS cells) (Figure 2a). MYOD-hAFS cells grew more slowly than EV-hAFS cells. The doubling time of MYOD-hAFS cells was nearly twofold longer than that of EV-hAFS cells in the myogenic media (data not shown). A longer doubling time suggests that MYOD-hAFS cells undergo myogenic differentiation because growth arrest is a prerequisite for cellular differentiation [18].

MYOD viral transduction induced morphological changes of hAFS cells into spindle shape from day 3. At day 10, a number of multinucleated myotube-like cells was observed. In contrast, no such myotube-like cells were observed in EV-hAFS cells (Figure 2a). The nuclei were crowded at both the center and the periphery in a multinucleated myotube-like cell. To confirm myogenic differentiation, we also examined myogenic marker expression in MYOD-hAFS cells during myogenic differentiation. Total RNA was collected on days 1, 3, and 7, and mRNA expression of *MYOG, MYH, MEF2C* and *DES* was analyzed using RT-PCR. MEF2C which activates myogenic transcription in complex with MYOD [27,28], was not enhanced by MYOD (Figure 2b). Desmin is expressed from an early stage of skeletal myogenesis [29-31]. Consistently, in hAFS cells transduced with MYOD-lentivirus, *DES* was expressed from day 1, peaked at day 3 and maintained up to day 7 (Figure 2b), suggesting that hAFS cells undergo skeletal myogenic differentiation by MYOD. *MYOG*, a gene expressed in the middle stage of skeletal myogenic development, is also expressed by MYOD from day 3 and the expression is increased up to day 7. *MYH,* a marker gene that distinguishes myogenic and non-myogenic differentiation, is also expressed at days 3 and 7 in MYOD-hAFS cells [32]. The results demonstrate that the expression of myogenic marker is consistent with morphological changes of hAFS cells into myotube-like cells by MYOD transduction.

### MYOD-hAFS cells express pre-myogenic and myogenic markers

To determine which pre-myogenic mesoderm factors can mediate myogenesis of hAFS cells expressing *MYOD*, RT-PCR was performed with total RNA collected on days 1, 3 and 7. Pre-myogenic mesoderm factors such as *MEOX1, PAX7, SIX1* and *EYA2* are expressed in the paraxial mesoderm and developing somite of the embryo [33]. PAX3 is temporarily expressed during an early proliferation period of myogenic progenitors [34,35], whereas PAX7 is expressed in non-proliferating and undifferentiated cells, and down-regulates MYOD [36]. In MYOD-hAFS cells, *PAX3* was up-regulated by *MYOD* at day 1 and then decreased until day 7 (Figure 2c). However, *PAX7* was not detected in MYOD-hAFS cells (data not shown). These results suggest that PAX7 does not mediate MYOD-induced myogenesis. In addition, the genes involved in limb muscle development (Meox1) [37], and PAX3 up-regulation (Six1 and Eya2) [38] were also up-regulated in *MYOD* transduced hAFS cells, compared to EV transduced cells (Figure 2c). MYOD induced expression of *SIX1*, *MEOX1* and *EYA2* at specific time periods. *SIX1* gene was decreased at day 1, but induced at day 3 by MYOD. *MEOX1* gene was induced very early on day 1 by MYOD. *EYA2* expression was induced by MYOD throughout the differentiation period. Expression of *GLI2*, *FOXC1* and *FOXC2,* which are involved in muscle deficiency [37,39-44], was not regulated by *MYOD* transduction (Figure 2c). Thus, in hAFS cells, *MYOD* transduction induced up-regulation of pre-myogenic mesoderm factors, such as *PAX3*, *MEOX1*, *SIX1* and *EYA2,* prior to up-regulation of myoblast marker gene expression.

To determine whether the protein levels are correlated with gene expression and morphological myogenic differentiation, western blot analysis was performed with anti-MYF5, -myogenin, -desmin, -dystrophin, and -MYOD antibodies on total protein extracted on days 1, 3, 7, and 10. As expected, MYOD protein was induced from day 1 and peaked at days 3 and 7 in MYOD-hAFS cells, but little was expressed in EV-hAFS cells (Figure 2d). MYF5 expression was not changed by MYOD transduction during myogenesis, whereas desmin was strongly expressed from day 3 (Figure 2d). Dystrophin, a major component of the dystrophin–glycoprotein complex in muscle fibers, responsible for the maintenance of sarcolemma integrity [45], was also expressed from day 3. Because desmin is an early marker of skeletal myogenic lineage differentiation and is expressed in myogenic committed-mononucleated cells before fusion [46], hAFS cells transduced with MYOD are differentiated into skeletal myoblasts.

### Intracellular localization of myogenic proteins and phosphorylation/dephosphorylation of MYOD in hAFS cells

**Figure 3:**
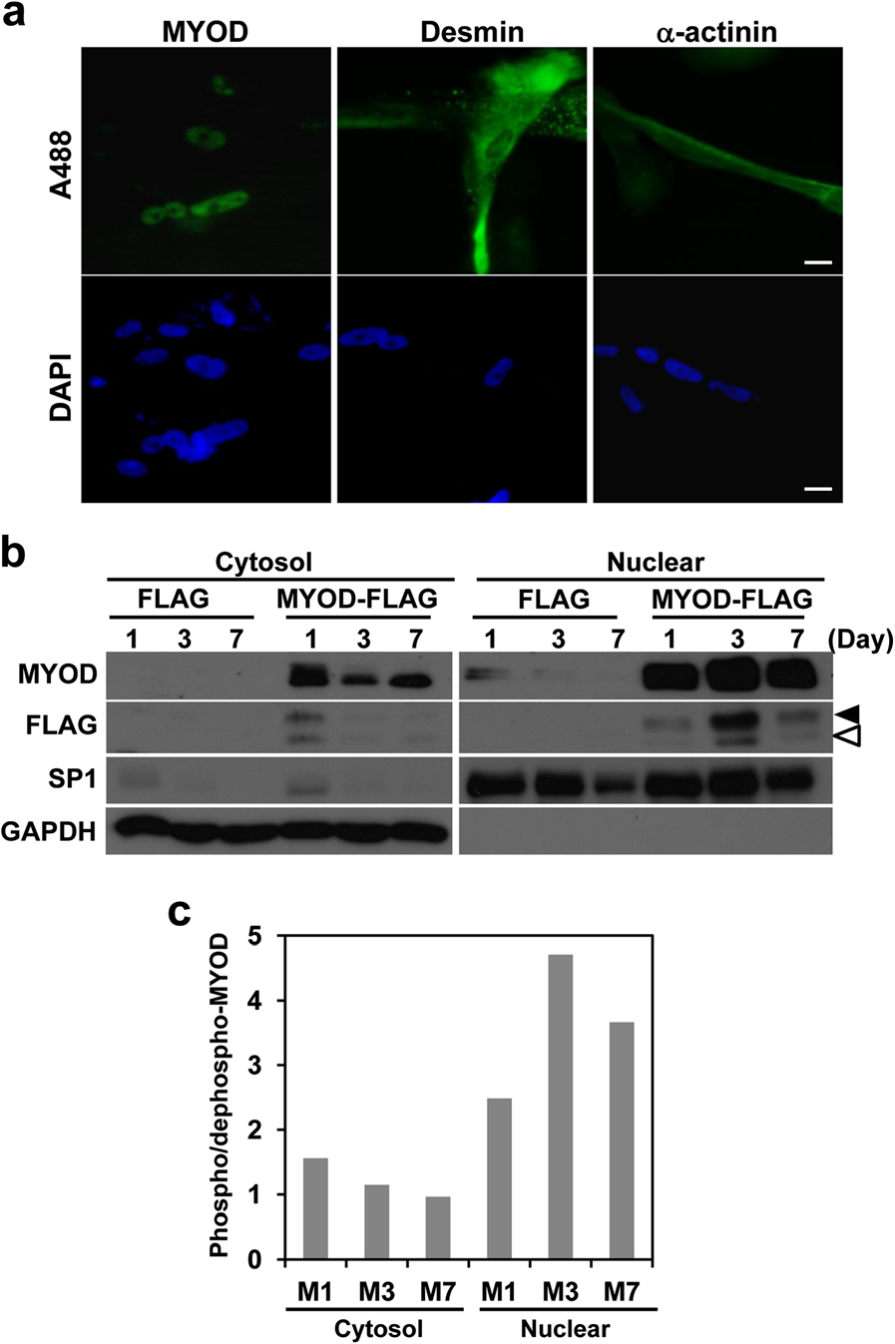
**Intracellular localization of MYOD, desmin and α-actinin proteins during myogenesis. (a)** Immunocytochemistry of hAFS cells infected with MYOD lentivirus. The infected cells were cultured in myogenic differentiation medium for seven days. Myogenic marker proteins, MYOD, desmin and α-actinin were immunostained with monoclonal antibodies and Alexa Fluor 488 conjugated secondary antibody (upper). Nuclei were stained with DAPI (lower). **(b)** Western blotting of MYOD protein in the nuclear-cytosol fractions of differentiating hAFS cells at 1, 3 and 7 days. hAFS cells were transduced with FLAG pHJ-1 (FLAG) or MYOD-FLAG pHJ-1 (MYOD-FLAG). Phosphorylated MYOD (black arrow head, 47 KDa) and dephosphorylated MYOD (white arrow head, 45 KDa) are indicated. **(c)** The ratio of phosphorylated and dephosphorylated MYOD during myogenic differentiation. The band densities of Flag tagged MYOD at **(b)** were measured and the densities were calculated as phosphorylated/dephosphorylated MYOD (Scale bar = 10 μm). DAPI, 4',6-diamidino-2-phenylindole; hAFS, human amniotic fluid stem.

To further confirm myogenic differentiation of hAFS cells by *MYOD* transduction, we examined intracellular localization of MYOD and myogenesis-related proteins. As expected, over-expressed MYOD was predominantly localized in the nucleus, suggesting that they are actively involved in expression of myogenesis-associated genes. In addition, desmin was also induced by MYOD transduction and localized in the cytoplasm of hAFS cells. α-Actinin, a marker for terminal myogenic differentiation, was detected only in myotubes (Figure 3a). These results suggest that MYOD transduction induces sufficient myogenic differentiation to the terminal stage for formation of myotubes.

To determine whether phosphorylation of MYOD is associated with myogenic differentiation of hAFS cells, the cells were transduced with FLAG tagged MYOD lentivirus. (Figure 3b) The cell pellet was fractionated into cytosol and nuclear fractions, as described in the Methods section, and each fraction was confirmed using nuclear specific SP1 protein and cytosol specific GAPDH protein. MYOD was expressed mainly as 47 kDa (phosphorylated form) [18] from day 1. At day 3, both phosphorylated and dephosphorylated MYOD reached their highest levels; however, phosphorylated MYOD was dominant over the dephosphorylated one (Figure 3b and c). Of particular interest, MYOD-hAFS cells started to fuse with one another from day 3 and the highest expression of MYOD was observed during this time period. The level of phosphorylated and dephosphorylated MYOD was also increased. These results suggest that an increase of phosphorylated and dephosphorylated MYOD in the nucleus may contribute to the formation of multi-nucleated myotube.

### The contribution of hAFS cells to regeneration of skeletal muscle injury *in vivo*

**Figure 4:**
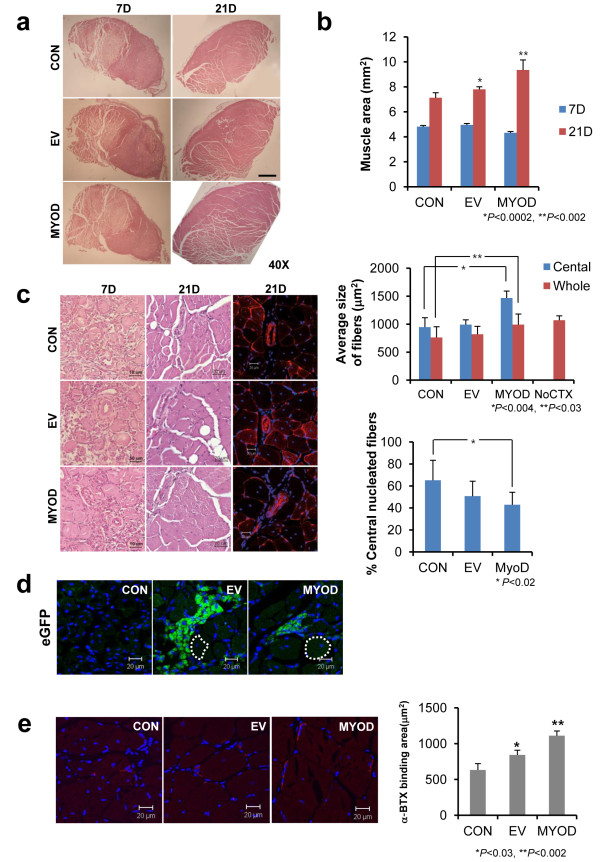
**Regeneration of TA muscle injury by MYOD transduced hAFS cells.** Left TA muscle of immunodeficient BALB/cSlc-nu mice was injured with cardiotoxin (0.4 nM/mouse), and EGFP positive hAFS cells transduced with EV and MYOD viruses. **(a)** H&E staining of TA muscles at 7 and 21 days after injection of EV- or MYOD-hAFS cells (black dashed line: injured site). **(b)** The area of muscle tissue was measured using the i-solution image program at 7 and 21 days after injection (n = 9; three per mouse). The muscle area was significantly increased in the MYOD-hAFS cell-injected group compared to the group injected with EV-hAFS cells or the control (*P <0.0002 and **P <0.002). **(c)** The size of muscle fibers of MYOD-hAFS cell-transplanted TA muscle was larger than that of CTX only or EV-hAFS cell injected TA muscles. Using the i-solution program, the average size of centrally nucleated myofibers and whole myofibers was calculated (n = 48; 16 per mouse) (*P < .004 and **P <0.03). The percentage of centrally nucleated fibers over total fibers was also calculated (n = 48; 16 per mouse) (*P <0.02). **(d)** Injected hAFS cells were visualized with eGFP signal (green) and DAPI with confocal microscopy (white dashed line: regenerating muscle fiber). **(e)** TA muscles injected with CTX only (control), EV- or MYOD-hAFS cells were stained with α-BTX at 21 days after injection. The area of α-BTX binding was measured using the i-solution image program (n = 32; 16 per mouse) (*P <0.03 and **P <0.02) (Scale bar; a = 500 μm, 7 days of c = 10 μm and others = 20 μm). α-BTX, α-bungarotoxin; CTX, cardiotoxin; DAPI, 4',6-diamidino-2-phenylindole; EV, empty vector; hAFS, human amniotic fluid stem; TA, tibialis anterior.

Having established that MYOD transduction in hAFS cells induces differentiation to skeletal myotubes, we next asked whether MYOD-expressing hAFS cells contribute or enhance myogenic regeneration after *in vivo* transplantation. To label hAFS cells, EGFP lentiviruses were transduced to hAFS cells, and eGFP-positive cells were isolated with FACS Aria (93.82% positive; Additional file 1: Figure S1). EGFP-positive hAFS cells were cultured for three days and transduced again with MYOD- or EV-lentiviruses. hAFS cells transduced twice with eGFP and MYOD or empty viruses were able to differentiate into myoblasts (data not shown). Transduction efficiency was measured with eGFP lentivirus (about 80%; Additional file 2: Figure S2). Transduction of MYOD lentivirus was confirmed with Western blot with MyoD antibody (Figure 2d). These eGFP-positive MYOD-expressing or eGFP-positive EV-expressing hAFS cells were co-injected with cardiotoxin (CTX) into TA muscles of immunodeficient mice (n = 3) [18,47]. CTX was injected to induce injury in the TA muscle [48]. After 7 and 21 days, the mice were sacrificed, and tissues were sectioned (5 μm) and stained with H&E. A total of 144 sections of each group (Control, EV-hAFS cell- and MYOD-hAFS cell-injected groups) were stained with H&E. Seven days after cell transplantation, no significant difference was observed among control, EV-, and MYOD-hAFS cell groups (Figure 4a). However, at 21 days after injection, muscle volume of the MYOD-hAFS cell-injected group was increased, compared to those of the control or EV-hAFS cell group (Figure 4a). At 21 days post injection, consistent with increased muscle volume, the muscle fiber area of MYOD-hAFS cell-transplanted TA muscle was larger than that of CTX only or EV-hAFS cell-injected TA muscles (n = 9) (Figure 4b). Forty eight sections in each group around the center area were used for immunofluorescence with dystrophin. The IHC images of dystrophin were used to calculate an average size of muscle fibers. IHC staining with dystrophin antibody also showed that muscle fibers of MYOD-hAFS cell-transplanted TA were larger than those of CTX only or EV-hAFS cell-injected TA muscles (Figure 4c). Histomorphometric analysis showed that the average size (μm^2^) of centrally nucleated myofiber was 947.9 ± 108.1 (control), 992.7 ± 85.7 (EV) and 1,467.9 ± 124.0 (MYOD) (CON:MYOD *P <0.004). These results suggest increased size of muscle fibers injected with MYOD-hAFS cells. The average size of whole myofibers was 762.3 ± 191.7 (control), 820.1 ± 141.4 (EV), and 992.5 ± 188.2 (MYOD) (CON:MYOD **P <0.03). Similarly, muscle fibers injected with MYOD-hAFS cells showed an increase in size. Because the average size of normal myofibers in the TA muscle (no CTX) was 1,072.0 ± 77.3, increased size (992.5 ± 188.2) of myofibers by MYOD-hAFS cells may not mean hypertrophy. It seems rather to be normal growth of myofibers during regeneration. In addition, the percentage of centrally nucleated fibers (regeneration index) was 65.2% (control, 1,020/1,564), 50.8% (EV, 740/1,457) and 42.9% (MYOD, 595/1,386) (CON:MYOD *P <0.02). These results show that MYOD-hAFS cells injected TA muscle undergo an active regeneration (Figure 4c).

We also asked the question whether injected hAFS cells become a direct part of the myofiber or secrete some growth factors to induce formation of large myofibers. EGFP labeled EV- or MYOD-hAFS cells were injected into TA muscles. After 21 days, the muscle tissues were cryostat sectioned and observed under fluorescence microscopy. As shown in Figure 4d, the injected cells were clustered, but did not become part of the myofiber. These results suggest that hAFS cells expressing MYOD may induce large myofibers through paracrine action of some growth factors.

To examine the question of whether muscles regenerated by MYOD-hAFS cells are functional, cryostat sections were stained with Alexa-594 conjugated α-bungarotoxin (*a*-BTX). *a*-BTX allows for the detection of the distribution of acetylcholine receptor, which is confined to the postsynaptic membrane of the neuromuscular junction [13].

*a*-BTX positive signal was more frequently and strongly detected in MYOD-hAFS cell-injected TA muscles than control and EV-hAFS cell-injected muscles (Figure 4e). For quantification, the *a*-BTX-bound area was measured using i-solution image software. The *a*-BTX-bound area was selected in the same location of five muscle slides showing the same position (each slide had eight TA muscle sections) and used for calculation of the average bounded area. The results showed that the *a*-BTX bound area in MYOD-hAFS cell injected muscle was 1.75 fold higher than that of the control, and 1.31 fold higher than that of the EV-hAFS cell injected muscle (Figure 4e). These results suggest that the neuromuscular junction of MYOD-hAFS cell injected muscle is regenerated.

## Discussion

Myogenic differentiation of hAFS cells can be induced by 5′-azacytidine (5′-AZA) in myogenic medium. 5′-AZA-stimulated hAFS cells express MYOD and some myogenic factors [7]. We also tested myogenic differentiation with DNA methyltransferase (DNMT) inhibitors. They only induced an early myoblast differentiation, but not myotube formation (data not shown). These results suggest that genetic reprogramming by DNMT inhibitor is not sufficient for induction of terminal myogenic differentiation of hAFS cells. On the other hand, expression of MYOD induces substantial myogenic differentiation of cells [49]. Consistent with these reports, forced expression of MYOD in hAFS cells also induced myogenic differentiation (Figure 2a and b). Results of myogenic marker analysis showed that late myogenic markers such as myogenin, MyHC and dystrophin were induced by forced expression of MYOD (Figure 2b), indicating that hAFS cells expressing MYOD can differentiate up to the late stage of myogenesis.

Muscle development is regulated by distinct molecular mechanisms. In extraocular, tongue and laryngeal muscles, and branchial arches, pituitary homeobox 2 predominantly controls the myogenic hierarchy, leading to up-regulation of MYF5 and MYOD, and, eventually, terminal differentiation induced by myogenin. In contrast, in limb muscles, SIX1 and EYA2 proteins regulate PAX3, which in turn controls proliferative myogenic cells [50]. Differentiation of myogenic cells is induced by a cascade involving MYF5, MRF4, MYOD and myogenin. In trunk muscles, MYF5 or MRF4 can exhibit parallel activation of MYOD and myogenin, whereas PAX3 acts upstream of MYOD [25]. Because forced expression of MYOD induced expression of pre-myogenic genes such as *PAX3*, *SIX1*, *MEOX1* and *EYA2*, myogenic differentiation of hAFS cells appears to occur through a mechanism similar to that of limb muscle development. These results suggest that MYOD directly or indirectly regulates expression of the pre-myogenic and myogenic genes. Recently Akizawa *et al*. showed that amnion-derived cells can be differentiated into myoblast [51]. We further examined in hAFS cells, MYOD lentivirus stimulated pre-myogenic and myogenic genes, and we also demonstrated regeneration of chemically injured muscle by MYOD expressing hAFS cells.

Transcriptional activity of MYOD is regulated by phosphorylation and dephosphorylation [16-19]. Over-expressed MYOD in hAFS cells was predominantly localized in the nucleus (Figure 3a and b), suggesting that they are actively involved in expression of myogenesis-associated genes. Analysis of the phosphorylation status of MYOD showed that the phosphorylated form of MYOD was dominantly detected at day 3, the time when fusion of hAFS cells starts to occur (Figure 3b and c). This is interesting because dephosphorylated MYOD causes cell fusion under conditions of high mitogenesis [17] and phosphorylation of Thr115 of MYOD negatively regulates mouse myoblast differentiation [25] through inhibition of the DNA binding activity of the MYOD homodimer [16]. Therefore, dephosphorylated MYOD may positively regulate myogenic differentiation.

However, during enhanced myogenic differentiation of hAFS cells by forced expression of MYOD, MYOD became highly phosphorylated at day 3 (Figure 3b). This appears to be contrary to previous reports [16,17,25]. However, MYOD overexpression induced an increase of both phosphorylated and dephosphorylated MYOD, and, thus, a relative amount of dephosphorylated MYOD was also increased, compared to control (Figure 3b). Therefore, an increase in the amount of dephosphorylated MYOD, rather than the phosphorylation/dephosphorylation ratio, may participate in myogenic transcription in hAFS cells expressing MYOD. Another possibility is that phosphorylated MYOD may form a heterodimer with other factors, such as E12 [16], and regulate myogenic differentiation.

Evaluation of the myogenic effect of forced MYOD expression *in vivo* is needed for determination of its effect n muscle regeneration. A previous study using hAFS cells reported that transplantation of unstimulated hAFS cells into injured TA muscle had no detectable effect on regeneration [12,52], although hAFS cells show an immunomodulatory effect [53] and recruit host progenitor cells that help in the regeneration of the injured region [54]. In this report, we transplanted hAFS cells overexpressing MYOD into injured TA muscle, and found that muscle volume and myofiber size were increased by hAFS cells expressing MYOD (Figure 4). The increased muscle volume appears to be due to increased myofiber size; however, increased size of myofiber did not look like hypertrophy. Because a significantly large number of hAFS cells were transplanted, we can assume that the injected cells differentiate and fuse with host myoblasts. This is the case in MSCs, showing that MYOD transduced MSCs fuse with myoblasts to form the terminal differentiated myofiber within eight to twelve weeks in a dog [55]. However, injected hAFS cells were maintained and clustered within muscle tissues and did not fuse with host myofiber at 21 days after transplantation (Figure 4d). This may be because injected hAFS cells did not have sufficient time to fuse with host muscle fiber. Of particular interest, even though transplanted hAFS cells did not fuse with host myofiber, hAFS cells expressing MYOD contribute significantly to the increased size of myofibers. Therefore, paracrine action of hAFS cells expressing MYOD may contribute to the increased size of myofibers. In addition, the paracrine factor may also regulate formation of the neuromuscular junction. Development of neuromuscular junction represents muscle functionality and this may also contribute to the increased size of myofibers. Currently, the nature of paracrine factor released from hAFS cells expressing MYOD is not known. However, it may control regeneration of myofiber.

## Conclusion

In conclusion, hAFS cells were shown to be effectively differentiated to myogenic lineage by the transduced MYOD. The underlying mechanism includes expression of pre-myogenic factors followed by myogenic proteins, leading to skeletal myogenesis. Subsequent *in vivo* study showed that MYOD-expressing hAFS cells enhance the size of myofibers, however, they do not fuse with host myofiber, indicating paracrine induction of myofiber formation. Thus, genetic modification and optimization of hAFS cells may provide a useful alternative tool for regeneration of damaged muscle, even dystrophic muscles.

## Abbreviations

CTX: Cardiotoxin; DAPI: 4′,6-diamidino-2-phenylindole, dihydrochloride; (D)MEM: (Dulbecco’s) modified Eagle’s medium; DNMT: DNA methyltransferase; DTT: Dithiothreitol; EDTA: Ethylenediaminetetraacetic acid; eGFP: Enhanced green fluorescent protein; ES-FBS: Fetal bovine serum, embryonic stem cell qualified; EV: Empty vector; FACS: Fluorescence-activated cell sorting; FBS: Fetal bovine serum; H&E: Hematoxilin and eosin; hAFS: Human amniotic fluid stem; HEPES: Hydroxyethyl piperazineethanesulfonic acid; IgG: Immunoglobulin G; MHC: Major histocompatibility; MSC: Mesenchymal stem cells; PBS: Phosphate buffered saline; RIPA: Radio immunoprecipitation assay; RT-PCR: Reverse transcription polymerase chain reaction; TA: Tibialis anterior; TRITC: Tetramethyl rhodamine isothiocyanate; α-BTX: α-bungarotoxin; α-MEM: α- Minimum essential media.

## Competing interests

The authors declare that they have no competing interests.

## Authors’ contributions

JAK designed and performed experiments, analyzed data and drafted the manuscript. YHS, JOL, JJY and HIS discussed the results and implications and commented on the manuscript over the stages. EKP conceived and administrated the experiments, and helped to draft the manuscript. All authors read and approved the final manuscript.

## Supplementary Material

Additional file 1: Figure S1 Confirmation of EGFP positive human AFS cells. Three to five days after eGFP lentivirus transduction, cells were trypsinized and analyzed with FACS. The results showed that 93.82% of the cells were positive for GFP. Click here for file

Additional file 2: Figure S2 Transduction efficiency of lentivirus. To examine lentivirus transduction efficiency, EGFP lentivirus was transduced to hAFS cells. After three days of culture, fluorescent cells were counted using i-solution program. The results showed that transduction efficiency of 1/2 diluted eGFP lentiviruses was about 80% (Scale bar = 10 μm). Click here for file
