# Supplementary figures and images for: MYOD mediates skeletal myogenic differentiation of human amniotic fluid stem cells and regeneration of muscle injury
Source: Stem Cell Res Ther. 2013 Dec 11;4(6):147. doi: 10.1186/scrt358 (PMC4054934; doi:10.1186/scrt358)

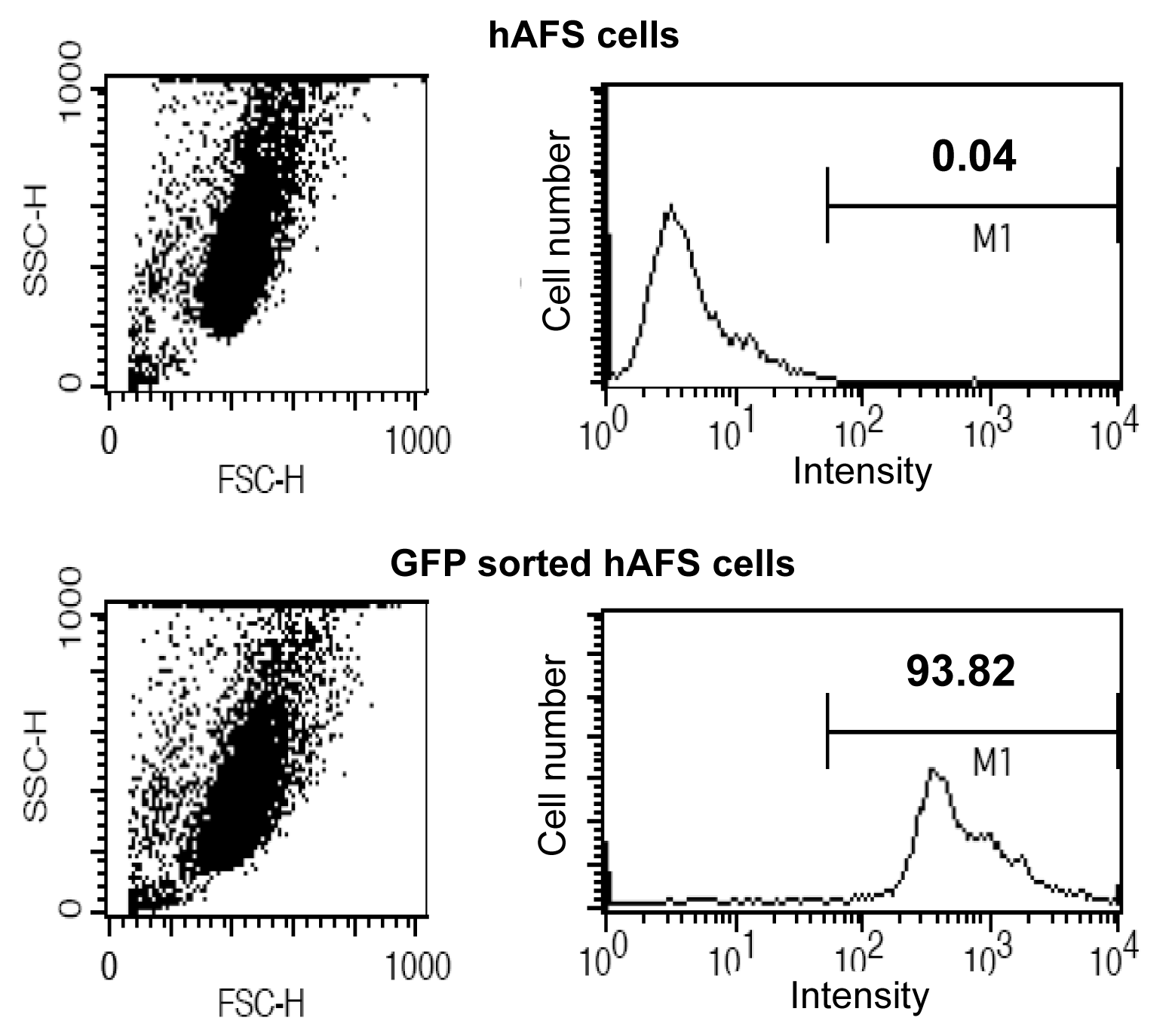

Supplement: Additional file 1: Figure S1 — Confirmation of EGFP positive human AFS cells. Three to five days after eGFP lentivirus transduction, cells were trypsinized and analyzed with FACS. The results showed that 93.82% of the cells were positive for GFP. [file scrt358-S1.tiff]

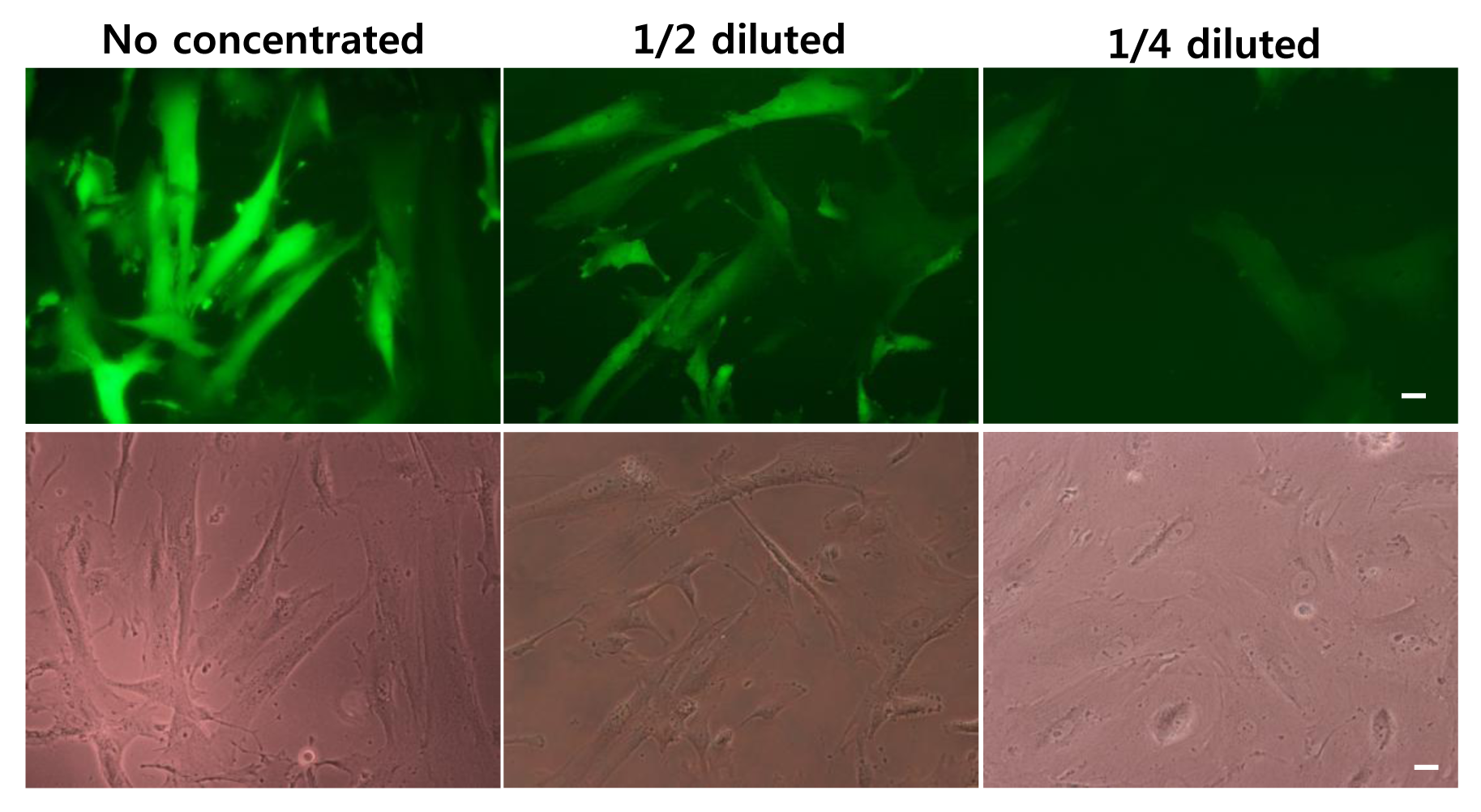

Supplement: Additional file 2: Figure S2 — Transduction efficiency of lentivirus. To examine lentivirus transduction efficiency, EGFP lentivirus was transduced to hAFS cells. After three days of culture, fluorescent cells were counted using i-solution program. The results showed that transduction efficiency of 1/2 diluted eGFP lentiviruses was about 80% (Scale bar = 10 μm). [file scrt358-S2.tiff]
